# A method for the quantitative determination of glycerophospholipid regioisomers by UPLC-ESI-MS/MS

**DOI:** 10.1007/s00216-018-1517-5

**Published:** 2018-12-22

**Authors:** Katharina Wozny, Wolf D. Lehmann, Manfred Wozny, Berna Sariyar Akbulut, Britta Brügger

**Affiliations:** 1 Heidelberg University Biochemistry Center (BZH), Im Neuenheimer Feld 328, 69120 Heidelberg, Germany; 2 German Cancer Research Center (DKFZ), Im Neuenheimer Feld 280, 69120 Heidelberg, Germany; 3 MassMap GmbH & Co. KG, Meichelbeckstraße 13a, 85356 Freising, Germany; 4 Department of Bioengineering, Marmara University, Kadikoy, 34722 Istanbul, Turkey

**Keywords:** Lipidomics, Glycerophospholipids, Position isomerism, Regioisomers, UPLC-MS/MS, Targeted MS/MS

## Abstract

Diacyl glycerophospholipids (GPs) belong to the most abundant lipid species in living organisms and consist of a glycerol backbone with fatty acyl groups in *sn-1* and *sn-2* and a polar head group in the *sn-3* position. Regioisomeric mixed diacyl GPs have the same fatty acyl composition but differ in their allocation to *sn-1* or *sn-2* of the glycerol unit. In-depth analysis of regioisomeric mixed diacyl GP species composed of fatty acyl moieties that are similar in length and degree of saturation typically requires either chemical derivatization or sophisticated analytical instrumentation, since these types of regioisomers are not well resolved under standard ultra-performance liquid chromatography (UPLC) conditions. Here, we introduce a simple and fast method for diacyl GP regioisomer analysis employing UPLC tandem mass spectrometry (MS/MS). This GP regioisomer analysis is based both on minor chromatographic retention time shifts and on major differences in relative abundances of the two fatty acyl anion fragments observed in MS/MS. To monitor these differences with optimal precision, MS/MS spectra are recorded continuously over the UPLC elution profile of the lipid species of interest. Quantification of relative abundances of the regioisomers was performed by algorithms that we have developed for this purpose. The method was applied to commercially available mixed diacyl GP standards and to total lipid extracts of *Escherichia coli* (*E*. *coli*) and bovine liver. To validate our results, we determined regioisomeric ratios of phosphatidylcholine (PC) standards using phospholipase A_2_-specific release of fatty acids from the *sn-2* position of the glycerol backbone. Our results show that most analyzed mixed diacyl GPs of biological origin exhibit significantly higher regioisomeric purity than synthetic lipid standards. In summary, this method can be implemented in routine LC-MS/MS-based lipidomics workflows without the necessity for additional chemical additives, derivatizations, or instrumentation.

**Electronic supplementary material:**

The online version of this article (10.1007/s00216-018-1517-5) contains supplementary material, which is available to authorized users.

## Introduction

Glycerophospholipids (GPs) are the ubiquitous building blocks of biological membranes in bacteria and eukaryotes. Different types and compositions of head groups and hydrocarbon chain(s) are the basis for the multitude of distinct GP classes and species, which contribute to the complexity of biological membranes.

In recent years, mass spectrometry–based lipidomics has made substantial progress in quantitative analysis of lipids [1]. Several hundreds of molecular lipid species can be identified in a single LC-MS/MS run by analyzing combined molecular weight and fragment ion data [2]. On top of this, approaches are available to determine in GPs double bond positions in the attached fatty acyl residues [3–6]. However, one feature that is usually not addressed is the regioisomerism in mixed diacyl GP species. Mixed GPs containing acyl groups A and B can occur as two regioisomers A(*sn-1*)/B(*sn-2*) and B(*sn-1*)/A(*sn-2*).

First attempts to discriminate between GP regioisomers made use of phospholipases that stereospecifically hydrolyze at either *sn-1* or *sn-2* position [7]. Quantification of the released lyso-GP species hereby allows deducing the relative abundance of each regioisomer in a GP of interest. Negative ion MS/MS of GPs results in a number of structure-specific fragmentations with the fatty acid anions being by far the most abundant fragments [8]. Importantly, using low collision energies, MS/MS spectra of PC and phosphatidylethanolamine (PE) usually show different abundances of fatty acid anions released from *sn-2* compared to those from *sn-1* [9, 10]. In detail, *sn-2/sn-1* fatty acyl fragment ion intensity ratios of 0.92 to 3.11 for 15 individual molecular species of PE were determined [11]. This feature has been used to establish calibration curves, correlating the composition of PC regioisomer mixtures to the *sn-2*/*sn-1* fatty acid anion fragment intensity ratio [12]. This concept, however, ideally requires the availability of a pure regioisomeric standard for each species. Due to the limited availability of such standards, this approach has not yet been systematically applied for regioisomer analysis. In another study, quantitative regioisomer analysis of PC was performed using the fatty acid ketene fragment that results from the loss of fatty acid-H_2_O as neutral species [13]. This ketene fragment exhibits a higher preference to be released from *sn-2* compared to the acyl anion fragment; however, its abundance is significantly lower. The analysis of synthetic PC regioisomer standards using this ketene-based fragment and other methods revealed regioisomeric purities of 75–96% [13]. In good agreement with these results, ozone- and collision-induced dissociation (CID)-MS showed regioisomeric purities of 77–91% of synthetic PCs [5].

Recently, advanced MS-based methods have been developed for the quantitative analysis of mixtures of regioisomers. One approach is based on baseline separation of regioisomers via differential mobility spectrometry (DMS) (for a review, see [14]). DMS introduces an additional ion separation step before the standard MS/MS analysis. Almost complete separation of the regioisomers PC 16:0/18:1 and PC 18:1/16:0 has been achieved using DMS, allowing to quantify their relative abundances [15]. A recent report describes selective detection of GP regioisomers by a hybrid MS^3^ approach. It employs CID followed by ultraviolet photodissociation to determine *sn* positions of fatty acyl chains via position-specific product ions [16]. In summary, a number of different approaches have been reported for GP regioisomer analysis. The most recent methods employ complex and advanced mass spectrometric instrumentation allowing regioisomer recognition by, e.g., specific gas-phase chemical reactions or laser-induced fragmentations. These advanced techniques generate regioisomer-specific fragment ions or achieve complete regioisomer separation before their analysis by MS/MS; however, the essential instrumentation is commonly not available in MS facilities.

Here, we focus on the exclusive use of regular LC-MS/MS instrumentation to determine GP regioisomer compositions, with a specific focus on regioisomer pairs that due to similar fatty acids attached are not well resolved under standard UPLC conditions. Based on targeted MS/MS combined with the new types of graphic and numeric data evaluation introduced here, we demonstrate the suitability of regular UPLC-MS/MS as a solitary technique for determining the regioisomeric composition of GP standards and of GPs of biological origin.

## Materials and methods

### Lipid standards and chemicals

The following standards and total lipid extracts were purchased from Avanti Polar Lipids: 16:0/18:1 PC (8500457), 18:1/16:0 PC (850475), 18:0/18:1 PC (850467), 18:1/18:0 PC (850476), and PC bovine liver (840053). All solvents/chemicals were purchased from Sigma-Aldrich. The following solvents were used for UPLC analyses: chloroform (GC grade), water (LC-MS grade), acetonitrile (LC-MS grade), 2-propanol (LC-MS grade), and formic acid (purity ≥ 96%). UPLC solvents contained 10 mM ammonium formate.

### Lipid extracts from *E*. *coli*

*Escherichia coli* K12 TB1 cells were cultivated as liquid culture in LB medium until an OD_600nm_ of approximately 1 was reached. Cell pellets were washed three times with 155 mM ammonium bicarbonate, pH 8.0. Subsequently, cell pellets were resuspended in 155 mM ammonium carbonate, pH 8.0. Phosphate determination was done as previously described [17]. For lipid analyses, cells equivalent to ∼ 1500 pmol phospholipid were subjected to MTBE extraction [18].

### Sample preparation

All lipids were dried under a nitrogen stream. Dried lipids were redissolved in 60% solvent A (60% acetonitrile/40% H_2_O, each containing 10 mM NH_4_HCO_2_ plus 0.1% formic acid) and 40% solvent B (90% 2-propanol/10% acetonitrile, each containing 10 mM NH_4_HCO_2_ plus 0.1% formic acid). Dissolution of ammonium formate in the two solvents was accomplished by sonication and thorough shaking. The samples were dissolved by vortexing for 2 min and subsequent sonication for 2 min. All lipid solutions were transferred to silanized glass inserts placed in Eppendorf tubes and then centrifuged at 6000×*g* for 2.5 min. Lipid concentrations ranging from 1 to 10 μM were used.

### UPLC-ESI-MS/MS analysis

Analyses were performed using an Ultimate^®^ 3000 LC system (Dionex, Thermo Fisher Scientific) with an ACQUITY UPLC CSH C18 1.7 μm, 1.0 × 150 mm column (Waters). The column oven temperature was set to 55 °C, that of the autosampler to 20 °C. The flow rate used was 100 μL/min. The starting solvent composition was as follows: 60% solvent A, 40% solvent B. The gradient was designed as follows: 0 min, 40% solvent B; 3 min, 50% solvent B; 9 min, 54% solvent B; 9.1 min, 70% solvent B; 17–22 min, 90% solvent B; and 22.5–30 min, 40% solvent B. MS analyses were performed in the negative ion mode using a Q Exactive instrument (Thermo Scientific). PC and PE molecules were detected as [M + HCOO]^−^ ions. The following ESI source parameters were used: sheath gas flow rate, 4; auxiliary gas flow rate, 0; sweep gas flow rate, 0; spray voltage, 4 kV; capillary temperature, 320 °C; S-lens RF level, 50. For full scans, the following parameters were employed: resolution, 35,000 at *m*/*z* 200; AGC target, 1e6; maximum IT, 200 ms; scan range, *m*/*z* 150–1500. Targeted MS/MS scans of [M + HCOO]^−^ adducts were performed in negative ion mode as follows: default charge, 1; resolution, 17,500 at *m*/*z* 200; AGC target, 2e5; maximum IT, 100 ms; isolation window precursor ions, 2.0 *m*/*z*; fixed first mass, 100 m/z; normalized collision energy (NCE), 25%. Targeted MS/MS scans were recorded with an average sampling frequency of 12 Hz.

### PLA_2_ digestion

Phospholipase A_2_ from porcine pancreas was purchased from Sigma-Aldrich (P6534, ≥ 600 U/mg). For the digestion, 200 pmol of lipid standards was pipetted into 2-mL Eppendorf tubes and dried under a gentle nitrogen stream. Dried lipids were resuspended in a mixture of 490 μL PBS buffer (Sigma-Aldrich, D8662) and 10 μL 100 mM CaCl_2_. The mixture was thoroughly vortexed (2 min at 450×*g*). Subsequently, 1 μL of the enzyme solution was added and the mixture was incubated at 37 °C for 4 h on a thermo block. Lipids were extracted by adding 500 μL of chloroform and 500 μL of methanol. The mixture was vortexed for 2 min at 450×*g* and centrifuged at 450×*g* for 3 min (at 4 °C). The bottom phase was transferred into a glass vial and dried under a gentle nitrogen stream. Lipid analysis by UPLC-ESI-MS was performed as described above, monitoring the peak areas of the lyso-lipids generated by PLA_2_ digestion.

### Data evaluation using MassMap

For data evaluation, Xcalibur raw files were converted into mzXML files using the open-source software *ProteoWizard* [19] using the MS convert option. For the conversion, the following parameters were applied: output format, mzXML; binary encoding precision, 32-bit; activation of check boxes “write index” and “TPP compatibility”; filters, MS level = 1. The resulting mzXML files were converted to MMP files (MassMap file format) using the corresponding file conversion software module. Full MS and MS/MS scans were separated by the software tool “extraction of full scans from MMP files,” followed by an automated search for the indicated molecules in a LIS file (TXT file containing the monoisotopic and average masses of molecules). Fragment ions were automatically assigned by the software and finally verified by the user.

### Computational-based quantitative determination of regioisomer ratios

As the first step in data deconvolution, the time interval containing the data points to be considered (fit interval) and the minimum relative intensity of the selected ion chromatograms (SICs) of the released fatty acids (quotient limit) were selected. The relative intensities were normalized to the maximum intensities of the respective peaks. The quotient limit was used for the construction of a quotient chromatogram (see below for definition). Only those data points above the quotient limit of the two [FA]^−^ SICs were considered. The two [FA]^−^ intensities recorded result from collision-induced release of fatty acids from *sn-1* and *sn-2* position of the GP of interest. If a GP of interest, for example a PC species containing fatty acids 16:0 and 18:1, is not regioisomerically pure, fatty acid 16:0 will be release from *sn-1* position in the one regioisomer and from *sn-2* in the other regioisomer, and vice versa. Two SICs will be recorded, one for fatty acid 16:0 and the other for fatty acid 18:1.

The procedure is based on the following set of data:

- Data points (*t*^P^_*i*_, *y*^P^_*i*_) with *i* = 1,2,…,*N*_P_. The numbers *t*^P^_*i*_ and *y*^P^_*i*_ represent the time and the intensity values of the *N*_P_ data points of the SIC chromatogram of the lipid precursor ions within the fit interval.
- Data points (*t*^*F*1^_*i*_, *y*^*F*1^_*i*_) with *i* = 1,2,…,*N*_F1_ and (*t*^F2^_*i*_, *y*^F2^_*i*_) with *i* = 1,2,…,*N*_F2_. The numbers *f*^F1^_*i*_, *y*^F1^_*i*_, *f*^F2^_*i*_, and *y*^F2^_*i*_ are selected in the same way as described in the previous paragraph for the SIC of the precursor ions. F1 and F2 denote the fragment ions of fatty acids 1 and 2, respectively.
- Data points (*t*^Q^_*i*_, *q*_*i*_) with *i* = 1,2,…,*N*_Q_. The numbers *t*^Q^_*i*_ stand for the time values of the data points of the fatty acid SICs. The numbers *Q*_i_ are calculated as *Q*_i_ = *y*^F1^_*i*_/*y*^F2^_*i*_. In short, each recorded data point is assigned to a specific time value.

The base peak functions *f*_1_(*t*) and *f*_2_(*t*) fitted to the chromatographic peaks are bell-shaped peaks. The shape is determined by a number of parameters (see Eqs. 1–3).

1 $$ {f}_i(t)=\exp \left(-{\left[\mathrm{l}\;t-{t}_{\max, i}\;\mathrm{l}/{W}_{\mathrm{e}\mathrm{ff}}\right]}^{{\mathrm{e}}_{\mathrm{e}\mathrm{ff}}}\right)\kern0.15em \mathrm{with}\kern.35em i=1,2 $$

2 $$ {\displaystyle \begin{array}{l}a\Big)\kern0.36em {w}_{eff}=\frac{h_{a,i}\cdot {w}_a+{h}_{b,i}\cdot {w}_b}{h_{a,i}+{h}_{b,i}}\\ {}b\Big)\kern0.36em {e}_{eff}=\frac{h_{a,i}\cdot {e}_a+{h}_{b,i}\cdot {e}_b}{h_{a,i}+{h}_{b,i}}\end{array}} $$

3 $$ {\displaystyle \begin{array}{l}a\Big)\kern0.36em {h}_{a,i}=\frac{1}{1+\exp \left(2\cdot \left(t-{t}_{\max, i}\right)/{w}_a\right)}\\ {}b\Big)\kern0.36em {h}_{b,i}=\frac{1}{1+\exp \left(2\cdot \left({t}_{\max, i}-t\right)/{w}_b\right)}\end{array}} $$

The parameters *t*_max,1_ and *t*_max,2_ are the locations of the maxima of the peaks of regioisomer 1 and regioisomer 2, respectively. Both base functions have their maximum values of 1 for *t* = *t*_max,1_ and *t*_max,2_, respectively.

It is assumed that, apart from the slight chromatographic separation, the positional isomers exhibit the same chromatographic behavior. Hence, the base peak functions used for simulation have the same shape. The shape of the peaks is determined by the parameters *w*_a_ and *w*_b_ that determine the widths of the left and the right parts of the peaks, respectively. If equal values for *w*_a_ and *w*_b_ are preset, a constant width parameter applies. Otherwise, the effective width parameter *w*_eff_ changes from *w*_a_ to *w*_b_ when moving from *t* values well below *t*_max,*i*_ to *t* values well above *t*_max,*i*_. The change from *w*_a_ to *w*_b_ is brought about by the weighting functions *h*_a,i_ and *h*_b,i_. The *h*_a,i_ function varies smoothly from 1 to 0 when moving from *t* values well below *t*_max,*i*_ to *t* values well above *t*_max,*i*_, whereas the *h*_b,i_ function varies smoothly from 0 to 1 when moving from *t* values well below *t*_max,*i*_ to *t* values well above *t*_max,*i*_. The same method is used in order to smoothly change the effective exponent *e*_eff_ from *e*_a_ to *e*_b_ when moving from the left edge of the peak to the right edge of the peak. If, in addition to equal values for *w*_a_ and *w*_b_, the sum of parameters *e*_a_ and *e*_b_ would equal 2, the resulting peaks would be of Gaussian shape. By using different values for *w*_a_ and *w*_b_ as well as for *e*_a_ and *e*_b_, the method allows for the simulation of a large variety of more or less asymmetric bell-shaped peaks.

The least-squares fitting method used by the MassMap^®^ routine is of the steepest descent type. In addition to the determination of the peak shape parameters *w*_a_, *w*_b_, *e*_a_, and *e*_b_, it is employed to determine the locations *t*_max,1_ and *t*_max,2_ of the centers of the two peaks as well as the coefficients *c*_P,1_, *c*_P,2_, *c*_F1,1_, *c*_F1,2_, *c*_F2,1_, and *c*_F2,2_. Together with the functions *f*_1_(*t*) and *f*_2_(*t*), the coefficients define the fitted intensity values:

4 $$ {\displaystyle \begin{array}{c}\mathrm{a}\Big)\;{{y^{\mathrm{P}}}_i}_{,\mathrm{Fit}}={c}_{\mathrm{P},1}\cdot {f}_1\left({t^{\mathrm{P}}}_i\right)+{c}_{\mathrm{P},2}\cdot {f}_2\left({t^{\mathrm{P}}}_{\mathrm{i}}\right)\ \mathrm{with}\ i=1,2,\dots, {N}_{\mathrm{P}}\\ {}\mathrm{b}\Big)\;{{y^{\mathrm{F}1}}_i}_{,\mathrm{Fit}}={c}_{\mathrm{F}1,1}\cdot {f}_1\left({t^{\mathrm{F}1}}_i\right)+{c}_{\mathrm{F}1,2}\cdot {f}_2\left({t^{\mathrm{F}1}}_{\mathrm{i}}\right)\ \mathrm{with}\ i=1,2,\dots, {N}_{\mathrm{F}1}\\ {}\mathrm{c}\Big)\;{{y^{\mathrm{F}2}}_i}_{,\mathrm{Fit}}={c}_{\mathrm{F}2,1}\cdot {f}_2\left({t^{\mathrm{F}2}}_i\right)+{c}_{\mathrm{F}2,2}\cdot {f}_2\left({t^{\mathrm{F}2}}_{\mathrm{i}}\right)\ \mathrm{with}\ i=1,2,\dots, {N}_{\mathrm{F}2}.\end{array}} $$

The following sum of squared residuals is minimized in order to simultaneously fit the three chromatographic peaks (SIC peak of the precursor ions and [FA]^−^ SIC peaks) and the quotient values:

5 $$ {\displaystyle \begin{array}{l} Chisquare:= \sum \limits_{i=1}^{N_P}{\left(\frac{y_{i, Fit}^P-{y}_i^P}{\max \left({y}^P\right)}\right)}^2+\sum \limits_{i=1}^{N_{F1}}{\left(\frac{y_{i, Fit}^{F1}-{y}_i^{F1}}{\max \left({y}^{F1}\right)}\right)}^2+\sum \limits_{i=1}^{N_{F2}}{\left(\frac{y_{i, Fit}^{F2}-{y}_i^{F2}}{\max \left({y}^{F2}\right)}\right)}^2+3\cdot \sum \limits_{i=1}^{N_Q}{\left(\frac{y_{i, Fit}^{F1}}{y_{i, Fit}^{F2}}-{q}_i\right)}^2\\ {}\kern1.92em \max \left({y}^X\right):= \mathrm{maximum}\ \mathrm{of}\ \mathrm{the}\ \mathrm{values}\ {y}_1^X,{y}_2^X,...,{y}_{N_X}^X\kern0.24em \left(X=P,F1,F2\right).\end{array}} $$

The division by the values max(*y*^*X*^) is done in order to make the contributions of the three fitted peaks similar. Multiplication of the last sum of squared residuals by 3 leads to improved reliability of the fitted coefficients.

If the *sn-2*/*sn-1* fatty acid anion intensity ratio is known for the earlier eluting regioisomer, the fitting may be restricted to one of the coefficients, *c*_F1,1_ or *c*_F2,1_, whereas the other coefficient is calculated from the known yield ratio *Q*_1_ = *c*_F1,1_/*c*_F2,1_. If the relative ion yield of the two fatty acid fragments is known for the later eluting regioisomer, the known yield ratio *Q*_2_ = *c*_F1,2_/*c*_F2,2_ may be used in the same way for the determination of the coefficients *c*_F1,2_ and *c*_F2,2_. Otherwise, the fitted coefficients may be used to experimentally determine the yield ratios *Q*_1_ and *Q*_2_.

Based on this deconvolution, a mean value for the measured *sn-2*/*sn-1* fatty acid anion intensity ratio, defined as *R*_exp_ value, can be determined. In addition, with known *sn-2*/*sn-1* fatty acid anion intensity ratio of a 100% pure regioisomer (defined as *R*_pure_ value), the algorithm allows to quantitatively determine the purity of a selected GP regioisomer, defined as RI_%_ value, i.e., an RI_%_ value of 80% indicates the presence of 20% of the corresponding regioisomer.

## Results and discussion

To establish the quantitative determination of GP regioisomers, we chose the commercially available standards PC 16:0/18:1 and PC 18:1/16:0, which have been used repeatedly as benchmark for regioisomeric analyses [5, 13].

### Determination of regioisomer ratios by PLA_2_ digestion

We first investigated regioisomeric purity of the two PC standards using an established method, namely by PLA_2_ digestion and quantitative analysis of the resulting lyso-PC species [13, 20–22]. PLA_2_ hydrolyzes the *sn-2* ester bond with high specificity, resulting in the formation of free fatty acids and the corresponding lyso-glycerophospholipid species [7]. For the standard PC 16:0/18:1, we found the opposite regioisomer PC 18:1/16:0 to contribute with 16.4%, while the standard PC 18:1/16:0 contained 11.9% of its opposite regioisomer (Table 1). These results are in good agreement with data reported in the literature [5, 13]. Thus, although both PC standards are 100% chemically pure, as determined by thin-layer chromatography, gas chromatography of fatty acid methyl esters, and MS analysis [23, 24], they contain a certain fraction of the opposite regioisomer.

**Table 1:** Regioisomeric purities for PC standards and corresponding PC species from a bovine liver extract. Regioisomeric purity values were obtained by PLA_2_ digestion, by ratio calculation of integrated selected ion current chromatogram of fatty acid anions (manually), and by the *Fragmentation Analysis* module of the software MassMap (automated). All values correspond to mean ± standard deviation and are the results of quadruplicate measurements. *Std.*, standard; *n.a.*, not applicable

Analyte	Regioisomer (*sn-1/sn-2*)	Regioisomeric purity (%) PLA_2_	Regioisomeric purity (%) manually	Regioisomeric purity (%) MassMap
Std. PC 16:0/18:1	16:0/18:1	83.6 ± 1.1	84.7 ± 0.1	82.5 ± 1.3
	18:1/16:0	16.4 ± 1.1	15.3 ± 0.1	17.5 ± 1.3
Std. PC 18:1/16:0	18:1/16:0	88.1 ± 1.0	86.5 ± 0.1	88.5 ± 1.1
	16:0/18:1	11.9 ± 1.0	13.5 ± 0.1	11.5 ± 1.1
Liver PC 16:0/18:1	16:0/18:1	n.a.	97.8 ± 0.2	95.9 ± 1.0
	18:1/16:0	n.a.	2.2 ± 0.2	4.1 ± 1.0
Std. PC 18:0/18:1	18:0/18:1	92.5 ± 1.6	90.9 ± 0.2	88.7 ± 0.8
	18:1/18:0	9.5 ± 1.6	9.1 ± 0.2	11.3 ± 0.8
Std. PC 18:1/18:0	18:1/18:0	86.1 ± 1.0	88.4 ± 0.01	89.7 ± 1.1
	18:0/18:1	13.7 ± 1.0	11.6 ± 0.01	11.3 ± 1.1
Liver PC 18:0/18:1	18:0/18:1	n.a.	99.9 ± 0.1	99.9 ± 0.1
	18:1/18:0	n.a.	0.1 ± 0.1	0.1 ± 1.0

### UPLC separation of phospholipid regioisomers

**Fig. 1:**
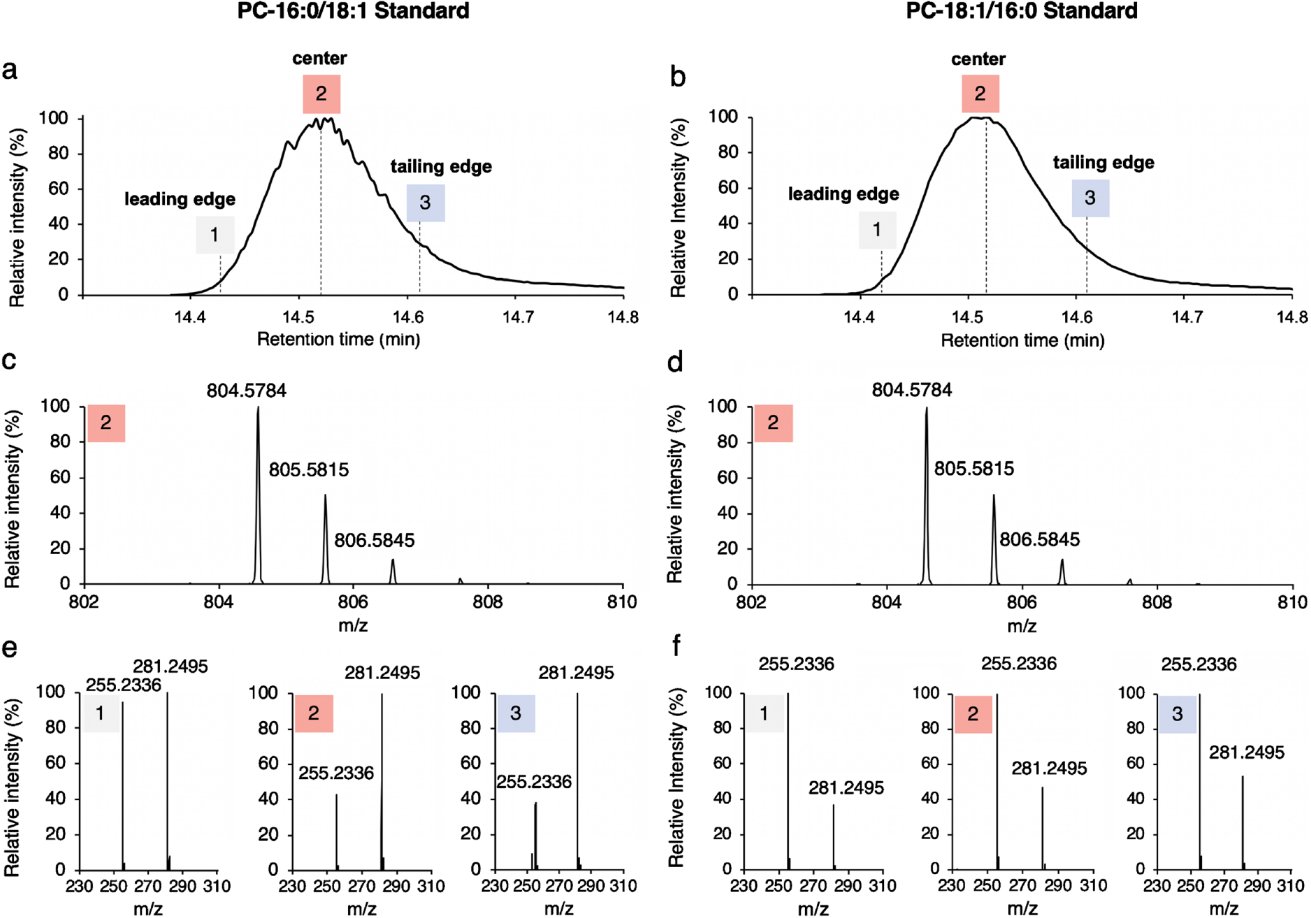
UPLC-MS/MS analysis of PC 16:0–18:1. **a**, **b** SIC of the [M + HCOO]^−^ adduct of PC 16:0/18:1 (**a**) and PC 18:1/16:0 (**c**). **c**, **d** MS1 spectrum recorded at the peak center (2) of the lipid elution profile shown in **a** and **b**, respectively. **e**, **f** MS/MS spectra of the [M + HCOO]^−^ precursor ion corresponding to the elution times 1, 2, and 3 indicated in **a** and **b**. *m*/*z* 255.2336 and 281.2495 correspond to the fatty acid anion of 16:0 and 18:1, respectively

We then performed targeted UPLC-MS/MS analyses of the two PC regioisomer standards, i.e., PC 16:0/18:1 and PC 18:1/16:0, with continuous data recording (alternating MS and targeted MS/MS scans) over the complete UPLC peak. Both lipids elute as a single peak as shown by SIC of the [M + HCOO]^−^ adduct (Fig. 1a, b) and molecular ion pattern (Fig. 1c, d). MS/MS analysis of the [M + HCOO]^−^ adduct at the indicated elution times covering the peak leading edge (elution time 1, Fig. 1a, b), the peak center (elution time 2, Fig. 1a, b), and the peak tailing edge (elution time 3, Fig. 1a, b) revealed different ratios of the two fatty acid anion fragments across the elution profile (Fig. 1e, f). Since the two regioisomers exhibit inverse intensity ratios of their fatty acid fragments (compare middle panels of Fig. 1e, f), the observed changes in the relative abundances of fatty acids 16:0 and 18:1 monitored at elution time points 1, 2, and 3 (Fig. 1e, f) indicate for both lipids the presence of the opposite regioisomer. These data also demonstrate that the regioisomer pair 16:0–18:1 (the hyphen indicates that the position of each fatty acid is not defined, while a dash specifies the fatty acid position to *sn-1* and *sn-2* [25]) is partially separated under standard LC conditions.

**Fig. 2:**
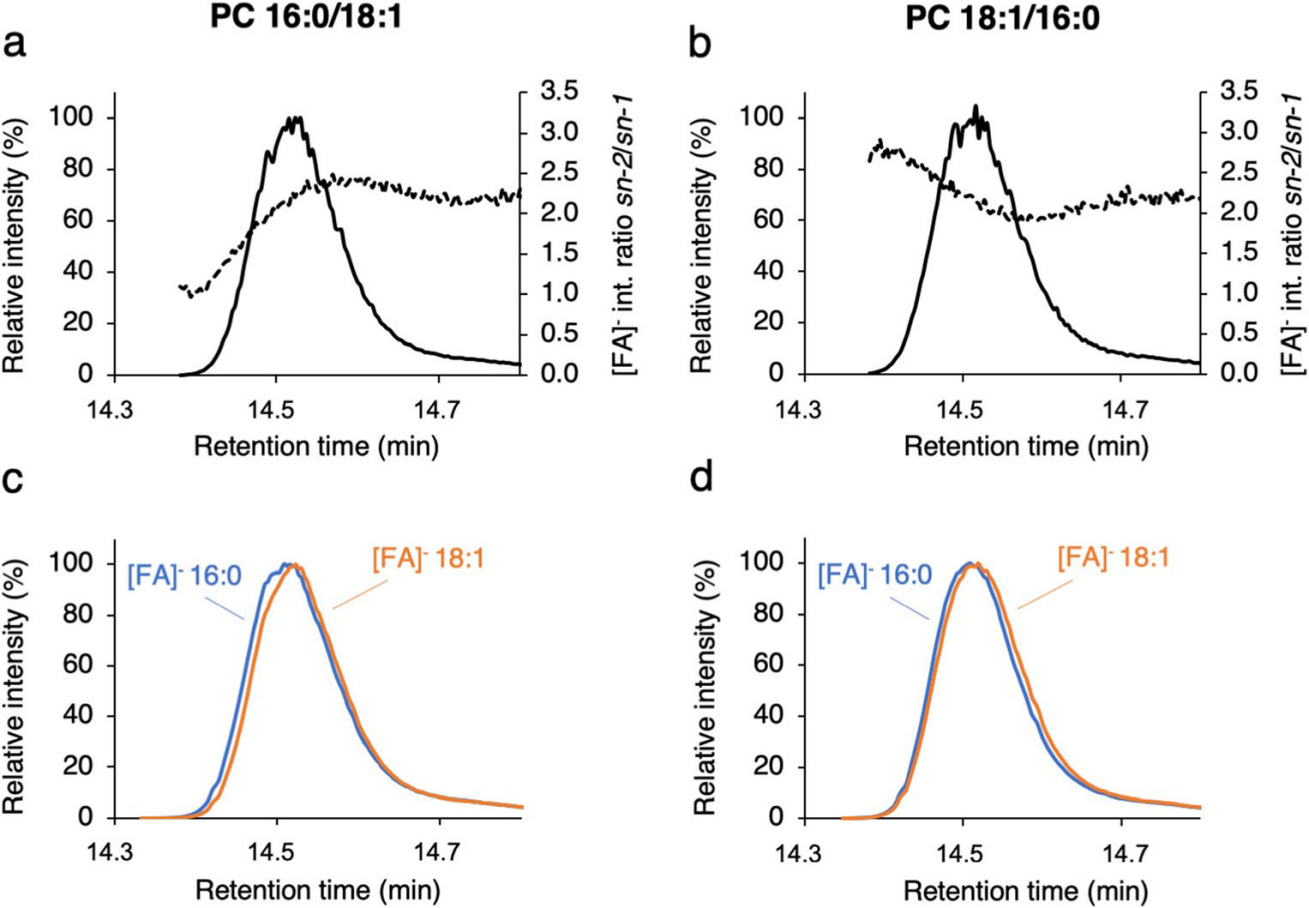
UPLC-MS/MS data for PC 16:0–18:1 standards. **a**, **b** Elution profile (SIC of the [M + HCOO]^−^adduct in full MS mode, line) and corresponding fatty acid ([FA]^−^) intensity ratios (*sn-2/sn-1*) (MS/MS mode, dotted line) for PC 16:0/18:1 (**a**) and PC 18:1/16:0 (**b**). **c**, **d** Elution profile (MS/MS mode, SIC) of the individual fatty acid anion fragments [FA]^−^ 16:0 at *m*/*z* 255 (blue) and [FA]^−^ 18:1 at *m*/*z* 281 (orange) of PC 16:0/18:1 (**c**) and PC 18:1/16:0 (**d**). Fragment ion SICs were normalized to 100% intensity before overlap display

By alternating recording of full MS and targeted MS/MS spectra of the [M + HCOO]^−^ ion over the elution profiles, more than 100 MS/MS and MS spectra, respectively, were acquired. Each MS/MS spectrum results in one intensity for each fatty acid anion (see Electronic Supplementary Material (ESM) Fig. S1). Their relative intensities can be displayed as *sn-2*/*sn-1* intensity ratios (Fig. 2a, b, dotted lines) over the elution profile together with the SIC of the precursor ion (Fig. 2a, b, solid lines).

Importantly, the assignment of *sn-2* and *sn-1* fatty acid is based on the “input” lipid, i.e., when analyzing PC 16:0/18:1, the 16:0 fatty acid anion with *m*/*z* 255 is assigned to the *sn-1* position. By displaying the SIC of the fatty acid anions at *m*/*z* 255 (16:0) and *m*/*z* 281 (18:1) as normalized overlay plots (Fig. 2c, d), we observed a minor but reproducible shift in elution times (each plot set to a relative intensity of 100%, non-normalized data are displayed in ESM Fig. S1): For both PC 16:0/18:1 and PC 18:1/16:0, the released fatty acid anion 16:0 elutes slightly earlier than the fatty acid anion 18:1 (Fig. 2c, d). Since the fatty acid in *sn-2* position gives rise to higher intensities, it can be concluded from this data that PC 18:1/16:0 elutes earlier than its regioisomer PC16:0/18:1. As visualized by the dotted line in Fig. 2a, at the leading edge of the elution peak of PC 16:0/18:1, the *sn-2*/*sn-1* intensity ratio of fatty acid anions is significantly lower than at the peak’s center and tailing edge. Here, the lower *sn-2*/*sn-1* intensity ratio of fatty acid anions is caused by the presence of the regioisomer PC 18:1/16:0. The intensity of the 16:0 fatty acid anion (here assigned to the *sn-1* position since PC 16:0/18:1 is the lipid subjected to the analysis) increases with increasing amount of PC 18:1/16:0 present in PC16:0/18:1: Its fragmentation efficiency from *sn-2* position is higher in the former species and thus gives rise to higher ion abundances than that of fatty acid 18:1 (see Fig. 1e, left panel), which in PC 18:1/16:0 is in the less favored *sn-1* position. The inverse effect is observed for the standard PC 18:1/16:0 (Fig. 1f).

**Fig. 3:**
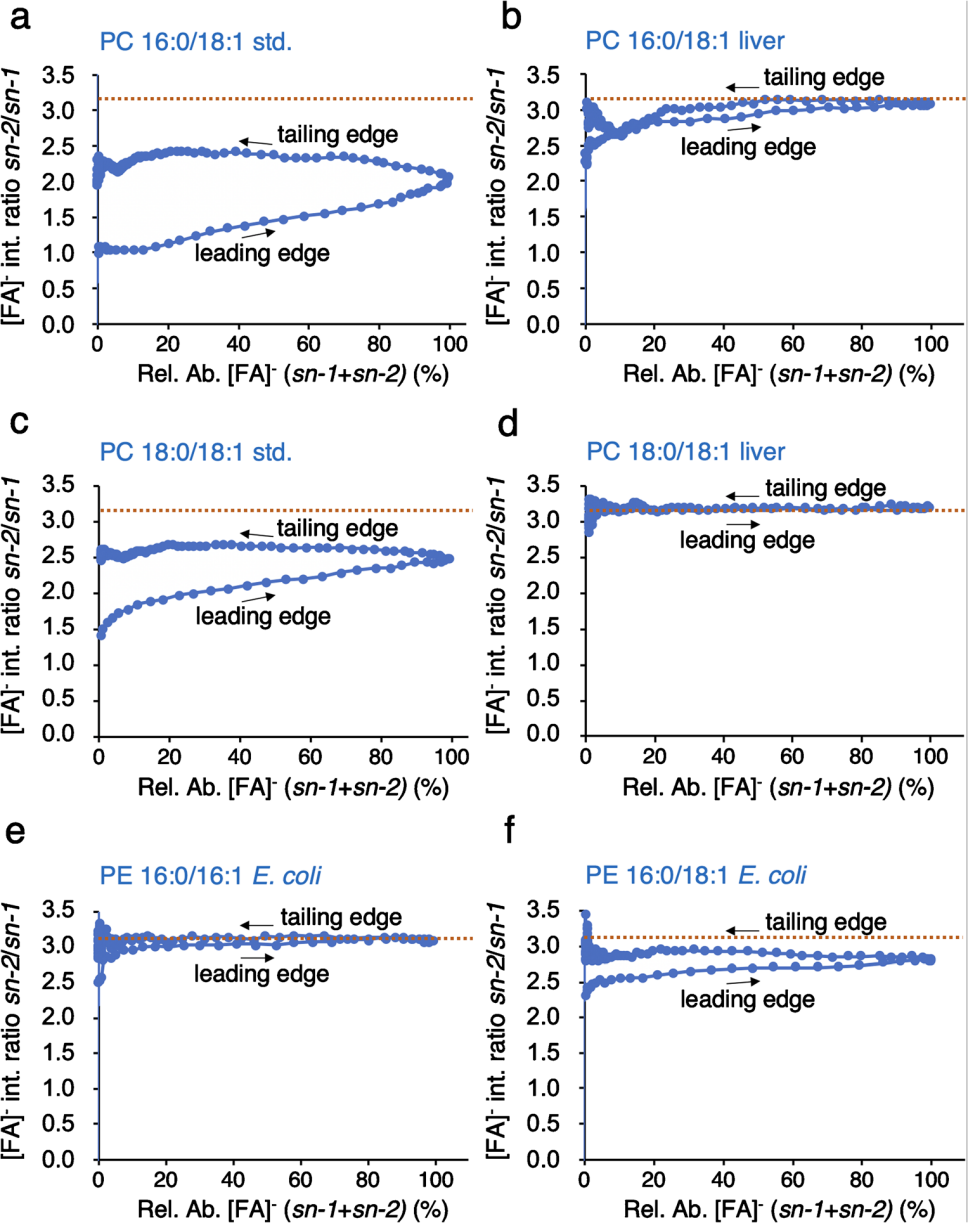
Regioisomeric purity plots for four selected PC species. **a** Standard PC 16:0/18:1, **b** bovine liver PC 16:0/18:1, **c** standard PC 18:0/18:1, **d** bovine liver PC 18:0/18:1, **e***E*. *coli* PE 16:0/16:1, and **f***E*. *coli* PE 16:0/18:1. The *R*_pure_ values of 3.18 (PC) and 3.19 (PE) apply to all analyzed PCs and PEs, respectively, and are displayed as dashed line (orange). Leading edges and tailing edges of chromatographic peaks are indicated by arrows

In order to visualize regioisomeric purity, we plotted the *sn-2*/*sn-1* [FA]^−^ fragment intensity ratio versus the relative abundance of the sum of both [FA]^−^ fragments (Fig. 3, for an exemplary raw data set, see ESM Fig. S2). This type of graph, here referred to as *regioisomeric purity plot*, correlates the experimentally determined *sn-2*/*sn-1* fragment ion intensity ratio with the elution profile of the analyte. The timeline of data recording is indicated by arrows at the leading edge and tailing edge of the UPLC peak, respectively. As a representative example, Fig. 3 shows regioisomeric purity plots for the two PC standards PC 16:0/18:1 and PC 18:0/18:1 (Fig. 3a, c) and their corresponding equivalents found in bovine liver extracts (Fig. 3b, d), and of two PE species from an *E*. *coli* extract (Fig. 3e, f). The regioisomeric purity plots show that the two PC species of biological origin exhibit a higher regioisomeric purity than do their synthetic cognates. The two indicators of regioisomeric composition are as follows: (i) the *sn-2*/*sn-1* ratio (from now on referred as *R*_exp_ value) and (ii) the spread of this ratio between the leading edge (LE) and tailing edge (TE) of the elution profile. Thus, the smaller the spread the regioisomerically purer is the compound investigated. Importantly, the regioisomer purity plot allows for the recognition of the fragment ion intensity ratio of a pure isomer (*R*_pure_) as is shown for liver-derived PC 18:0/18:1 and for *E*. *coli*–derived PC 16:0/16:1, where the data points of the leading and tailing edge almost coincide and result in an *R*_pure_ value of 3.18 and 3.19, respectively (Fig. 3d, e; see also ESM Fig. S3). Experimentally, we observed that this *R*_pure_ value is virtually identical for GP isomers within the same class, except for GPs containing polyunsaturated fatty acyl chains with double bond number ≥ 4. Figure 3 shows the same GP class-specific *R*_pure_ value for all species investigated (dotted line in Fig. 3). The *R*_exp_ value is proportional to the regioisomeric purity, i.e., higher ratio values indicate higher regioisomeric purity. These data demonstrate that plotting *sn-2*/*sn-1* intensity ratios of fatty acids against the elution time allows determining the relative contribution of each regioisomer.

### Computational determination of regioisomeric composition of GP species

**Fig. 4:**
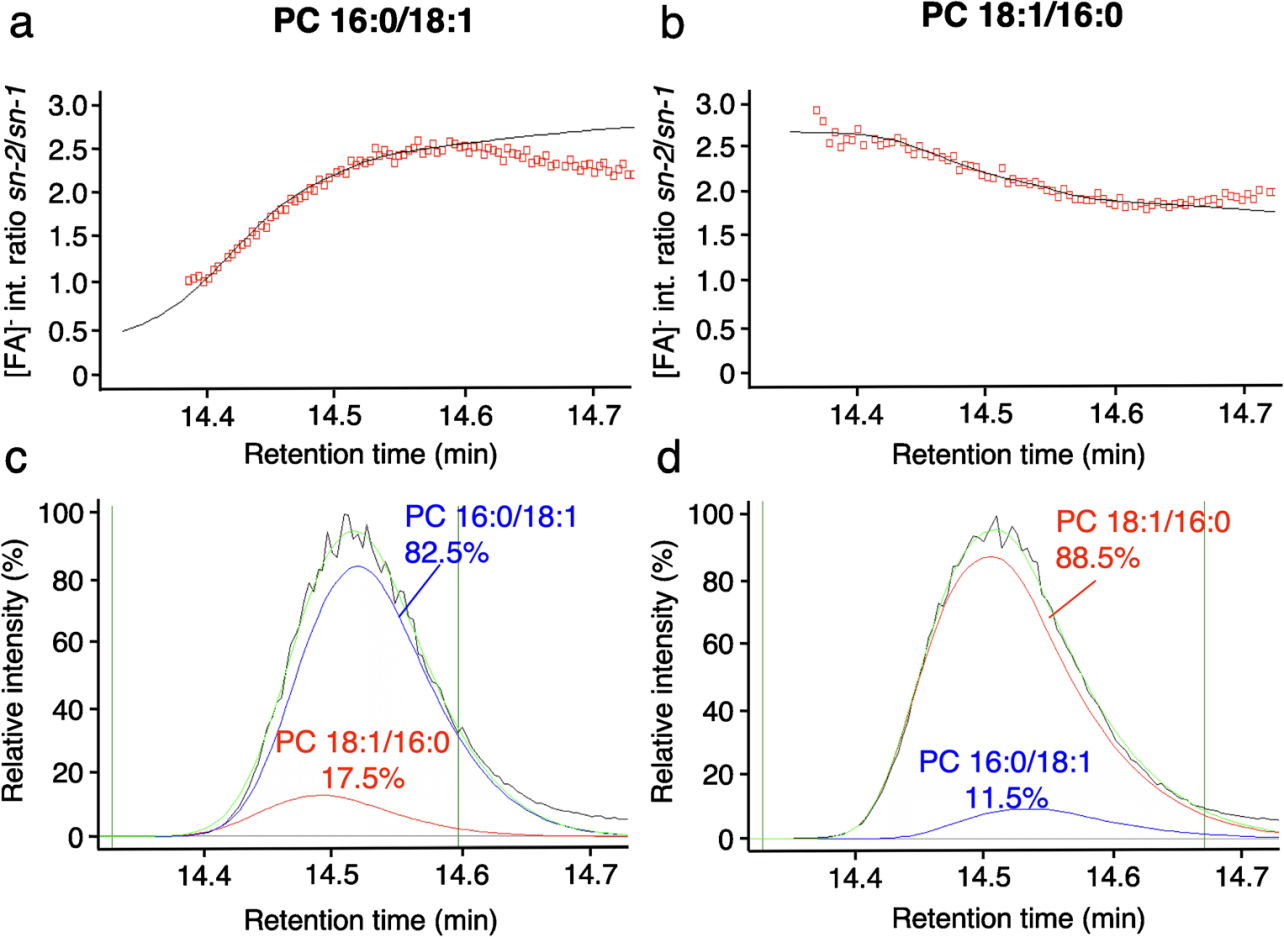
Experimental and reconstructed data for regioisomeric composition analysis by UPLC-MS/MS of the two standards PC 16:0/18:1 and PC 18:1/16:0. **a**, **b** Experimental fatty acid anion ([FA]^−^) *sn-2*/*sn-1* intensity ratios (open red squares) and exponential fit (black) for the standard PC 16:0/18:1 (**a**) and PC 18:1/16:0 (**b**). The fits in **a** and **b** are the basis for the reconstructed elution profiles of the individual regioisomers shown in **c** and **d**. **c** Experimental SIC of *m*/*z* 804.576 of the standard PC 16:0/18:1 (black) and three reconstructed elution profiles as follows: pure PC 16:0/18:1 (blue), pure PC 18:1/16:0 (red), and sum of both regioisomers (light green). **d** Experimental SIC of *m*/*z* 804.576 of the standard PC 18:1/16:0 (black) and three reconstructed elution profiles with color code as in **c**. Upper and lower time limits selected for data reconstruction are indicated by vertical lines (dark green)

To implement a computational determination of regioisomer relative abundances of GP standards and GPs of biological origin, we incorporated a *Fragmentation Analysis* module into the software MassMap. As a first step, an automated search of full scan data was implemented to assign GP molecular ion species. Subsequently, their fragment ions were assigned. *sn-2*/*sn-1* [FA]^−^ fragment intensity ratio plots were then calculated and a fit was applied to the ratio graph (fragment ion intensity ratio versus retention time). Representative results are shown for the standards PC 16:0/18:1 (Fig. 4a) and PC 18:1/16:0 (Fig. 4b).

By means of these fits, the individual elution profiles of the two regioisomer molecular species were reconstructed and their relative abundances were calculated. The results of the fits are shown for the PC standard 16:0/18:1 (Fig. 4c) and the PC standard 18:1/16:0 (Fig. 4d). With this reconstruction, the relative abundance of the major regioisomer (the regioisomeric purity, defined as RI_%_ value) was determined as 82.5% for PC standard 16:0/18:1 and 88.5% for PC standard 18:1/16:0. Thus, we observed the opposite regioisomer to be present with 17.5% and 11.5%, respectively, which is in good agreement with the literature [5, 13]. Mixtures of PC standard 16:0/18:1 and PC standard 18:1/16:0 at different molar ratios confirmed the regioisomeric purities as determined for the “pure” standard (see ESM Fig. S3). Besides the information on regioisomeric composition, MassMap also provides the retention time differences of the [FA]^−^ SIC profiles and of the reconstructed elution profiles of the individual regioisomers. The relative fragment ion abundances were reproducibly retained over a large concentration range, as shown for the regioisomeric composition of PC 34:1 from 0.02–20 μM (see ESM Fig. S4).

The regioisomeric composition of a given lipid can also be calculated manually based on the regioisomeric purity plots (as displayed in Fig. 3). Here, the calculations are done on the basis of the *R*_exp_ and the *R*_pure_ values, by introducing the term *H* that is defined as *H* = *R*_pure_/(1 + *R*_pure_). Hence, *H* represents the fraction of the major [FA]^−^ fragment intensity normalized to the sum of both fragment intensities. As an example, an *R*_pure_ value of 3 corresponds to an *H* value of 0.75. While *R*_pure_ is almost constant for each GP class, *R*_exp_ may be different for each GP sample, as it represents the weighted average of the *sn-2*/*sn-1* fragment intensity ratio. The weighted average of *R*_exp_ is obtained by calculating the ratio of the respective [FA]^−^ SIC peak areas (for an exemplary raw data set, see ESM Fig. S5). It is also one of the parameters determined by the newly established *Fragmentation Analysis* module in MassMap. To achieve reproducible integration results, SIC intensities were integrated within the limits of 2% at the leading edge and 5% at the tailing edge. With these parameters defined, Eq. 6 allows to calculate the regioisomeric purity in % (RI_%_).

6 $$ {\mathrm{RI}}_{\%}=\left(\frac{H+{R}_{\mathrm{exp}}\cdot H-1}{2H+2{P}_{\mathrm{exp}}\cdot H-{R}_{\mathrm{exp}}-1}\right)\cdot 100 $$

The basic assumption for establishing this equation is that within one pair of regioisomers (1 and 2), the *R*_pure_ value for regioisomer 1 is the inverse of the value for regioisomer 2. The isomeric purity data determined for PC standards and for liver PC species (see Fig. 3) were manually calculated by Eq. 6 and were compared to both the results obtained by PLA_2_ digestion and by automated MassMap data evaluation (Table 1).

Data are shown for PC standards and PC species from bovine liver, which were selected to allow for the direct comparison of regioisomeric purity between identical GP species. The PLA_2_ digestion method is only applicable to pure analytes since in complex mixtures, the digestion products, fatty acids, and 2-lyso GP species cannot be linked to an individual precursor molecule. Therefore, Table 1 contains PLA_2_ data only for the chemically pure standard compounds. All three methods deliver regioisomeric purity values for the four investigated standards that are identical within < 2% on an absolute basis. These results demonstrate that our approach delivers correct regioisomeric purity data. Moreover, our study shows that this approach is also capable to characterize PC and PE species present in complex mixtures such as total lipid extracts from bacteria or tissue. In this type of analysis, two bovine liver PC species were characterized and found to exhibit regioisomeric purities of 97.8% and 99.9%, respectively. On the basis of these data and those of other GP classes, we conclude that most GP species of biological origin exhibit a higher regioisomeric purity than commercially available standards.

By means of the method described here, regioisomeric purity analysis can be extended to other classes of GPs. Once an *R*_pure_ value has been determined, e.g., by analyzing a regioisomerically pure species of biological origin, this *R*_pure_ value can be applied to an GP species irrespective of its biological origin and of the sample complexity, as long as identical experimental conditions are applied. The method is particularly attractive since it can be performed on any regular UPLC-MS/MS system and does not require dedicated hardware modification.

## Electronic supplementary material

ESM 1 (PDF 5.11 kb)

ESM 2 (XLSX 622 kb)

ESM 3 (XLSX 376 kb)

ESM 4 (XLSX 25.1 kb)

ESM 5 (XLSX 16.6 kb)

ESM 6 (XLSX 537 kb)
