# Supplementary material for: A method for the quantitative determination of glycerophospholipid regioisomers by UPLC-ESI-MS/MS
Source: Anal Bioanal Chem. 2018 Dec 22;411(4):915–24. doi: 10.1007/s00216-018-1517-5 (PMC6338697; doi:10.1007/s00216-018-1517-5)
Supplement: Supplementary file 1 — (PDF 5.11 kb) [file 216_2018_1517_MOESM1_ESM.pdf]

Analytical and Bioanalytical Chemistry

Electronic Supplementary Material

A method for the quantitative determination of glycerophospholipid regioisomers by UPLC-ESI-MS/MS

Katharina Wozny, Wolf D. Lehmann, Manfred Wozny, Berna Sariyar Akbulut, Britta Brügger

Additional files available under 10.1007/s00216-018-1517-5
